# Early disseminated cutaneous Lyme disease with SIADH, transaminitis, and pancytopenia: A case report

**DOI:** 10.1177/2050313X251320186

**Published:** 2025-02-12

**Authors:** Louis Deschênes, Philippe Jutras, Janie Bujold

**Affiliations:** 1 Département de dermatologie, Centre hospitalier universitaire de Québec, Université Laval, Québec, QC, Canada; 2 Département de microbiologie médicale et infectiologie, CISSS du Bas-Saint-Laurent, Rimouski, QC, Canada; 3 Département de dermatologie, CISSS de la Gaspésie, Maria, QC, Canada

**Keywords:** Lyme disease, *Borrelia burgdorferi*, tick bite, SIADH, transaminitis, pancytopenia

## Abstract

Lyme disease is a tick-borne disease predominantly caused by *Borrelia burgdorferi* in Canada. Early disseminated disease is challenging to diagnose and requires treatment to prevent severe sequelae. We report the case of a 71-year-old woman who was admitted for a fever. Her blood tests showed a syndrome of antidiuretic hormone secretion, transaminitis, and pancytopenia. During hospitalization, the patient was noted to have an initial annular patch, which was followed by multiple diffuse erythematous patches. After a comprehensive medical history by a dermatology consultant highlighting an exposure to a tick bite, early disseminated disease was diagnosed, and a complete remission was obtained with Doxycycline. Dermatologists must maintain a high clinical suspicion for Lyme disease.

## Introduction

Lyme disease is predominantly caused by *Borrelia burgdorferi* and is increasingly encountered in Canada.^1^ The initial cutaneous presentation of this disease is subtle and can easily be missed. In this context, hematogenous spread can occur and cause disseminated Lyme disease, which has multiple presentations depending on the systems involved.^2^ We hereby describe a case of early disseminated Lyme disease presenting with multiple erythemata migrantia (MEM), syndrome of antidiuretic hormone secretion (SIADH), transaminitis, and pancytopenia.

## Case report

A 71-year-old Caucasian woman was admitted for fever, myalgias, and fatigue. Her relevant past medical history included peripheral artery disease, hypertension, and type 2 diabetes. She went on a trip to rural Quebec 12 days prior to the fever, where she only recalled a possible insect bite after specific questioning on the subject. The patient’s home was not located in an endemic zone for Lyme disease. However, the city in which her trip took place is known as an endemic area for this zoonotic disease.

**Figure 1.**
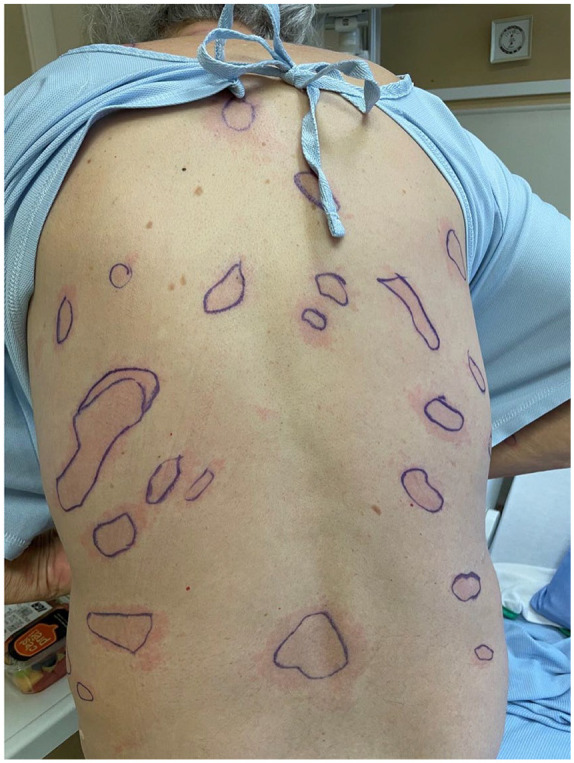
Erythematous oval and well-defined patches on the back.

**Figure 2.**
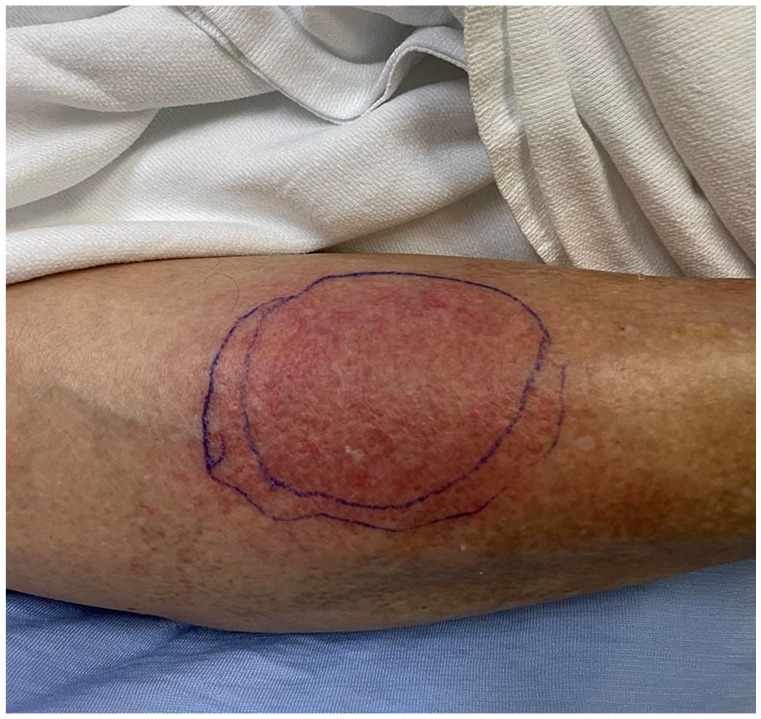
Erythematous and purpuric circular patch on the left lower leg.

**Figure 3.**
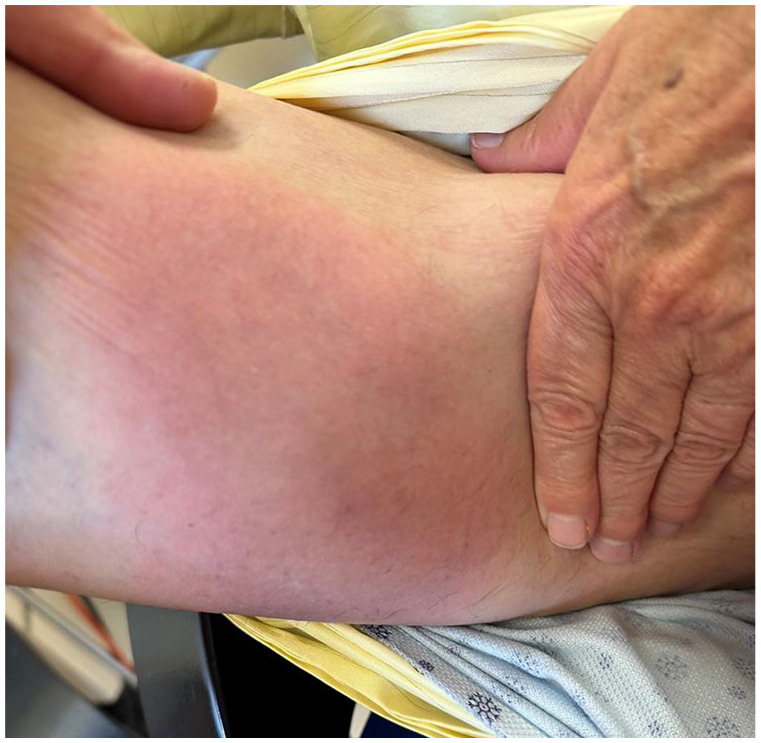
Erythematous and subtly annular patch with visible punctum on the right proximal leg.

At admission, fever was objectified, but other vital signs were within normal limits. Cardiorespiratory, musculoskeletal, neurological, and cutaneous examinations were stated as normal. A diagnostic of fever of unknown origin was made after a comprehensive infectious work-up came back negative. No antibiotics were prescribed at this stage. 15 days after the suspected insect bite, a red plaque was noted by the hospitalist on the right leg. The following day, multiple erythematous plaques were described on cutaneous examination. Three days later, a dermatology consult was ordered for this new skin eruption. Diffuse erythematous patches on the trunk, arms, and legs (Figures 1 and 2), including one on the right leg with a central punctum and an annular configuration (Figure 3), were noted.

Her most abnormal laboratory evaluation during her hospital stay showed sodium of 125 mmol/L (Normal (*N*): 134–143 mmol/L), C-reactive protein of 89 mg/L (*N*: 0–10 mg/L), white blood cells of 2.4 × 10^9^/L (*N*: 5.0–12.0 × 10^9^/L) with low levels of lymphocytes, hemoglobin of 109 g/L (*N*: 120–160 g/L), platelets of 85 × 10^9^/L (*N*: 140–440 × 10^9^/L), alanine aminotransferase (ALT) of 102 U/L (*N*: 11–54 U/L), and aspartate aminotransferase (AST) of 132 U/L (*N*: 15–41 U/L). Further testing showed serum osmolality of 274 mmol/kg (*N*: 280–301 mmol/kg), urine osmolality of 498 mmol/kg, and urine sodium of 63 mmol/L. SIADH was diagnosed and slowly corrected with fluid restriction and furosemide. An electrocardiogram detected a sinusal rhythm, associated with an absence of conduction disturbances. Serial troponin levels remained normal.

Her cutaneous eruption was biopsied, with histopathology showing a nonspecific dermis perivascular lymphoplasmohistiocytic infiltrate. Enzyme-linked immunosorbent assay for *Borrelia burgdorferi* antibodies was positive. This serologic test was made 19 days after the probable insect bite. Subsequent Western blot analysis immunoglobulin M was also positive. Immunoglobulin G was negative, showing an early infection of *Borrelia burgdorferi*. Polymerase chain reaction (PCR) test on fresh cutaneous tissue detected *Borrelia burgdorferi.* This test is not always required but was made in the context of suspected Lyme disease in a nonendemic area. No lumbar puncture was made, given the absence of neurological signs and symptoms. A search for coinfection with *Anaplasma phagocytophilum* remained negative, with tests such as search for morula on peripheral blood smear, PCR, and early and late serologies.

The patient was first clinically diagnosed, and then proved with paraclinical evaluation, with early disseminated Lyme disease, presenting with MEM, SIADH, pancytopenia, and transaminitis.

A treatment of Doxycycline 200 mg per day for 21 days was introduced with fast improvement. She was discharged 5 days later with the resolution of symptoms and normalization of her blood tests. The patient was seen in a follow-up 2 months later and had made a complete recovery.

## Discussion

*Borrelia burgdorferi* infection can present in three stages: early localized disease, early disseminated disease, and late disseminated.^3^ Early localized disease is characterized by erythema migrans, a marginated erythematous patch of at least 5 cm, sometimes with a central punctum, presenting 3–30 days after insect bite.^2^ This manifestation was probably overlooked in our patient before being observed during hospitalization. Even though there was a history of tick bites, it was not recalled by the patient at first and specific questioning in the context of progressive cutaneous lesions was needed. Indeed, tick bites are seldom recalled in patients with Lyme disease, complicating adequate care.^2^

Early disseminated disease is characterized by a hematogenous spread, which can present with neurologic symptoms, cardiac symptoms, and MEM.^3^ This cutaneous involvement generally presents itself days to weeks after initial erythema migrans, as multiple erythematous, symptomless, homogenous or annular, and well-defined oval patches.^2^ In our patient, those patches were noted quickly by the hospitalist at their onset.

SIADH is a frequent hypotonic euvolemic hyponatremia cause. SIADH’s causes include pulmonary diseases, malignancies, central nervous system disorders including infections, and drugs.^4^ Even though multiple cases have been described of Lyme disease presenting with SIADH secondary to neuroborreliosis,^5–7^ it is hereby impossible to make this diagnosis. The fact that no lumbar puncture was made and that no neurologic symptoms were present only allows us to view neuroborreliosis as a possibility. SIADH is well described in severe infections showing an absence of central nervous system involvement,^4^ but no Lyme disease case described corresponds to that description in our literature review.^5–7^ A lumbar puncture showing lymphocytic pleocytosis with a positive serology to *Borrelia burgdorferi* would have made neuroborreliosis the leading cause of SIADH in our patient.^8^ The decision was made with an infectious disease consultant to treat as cutaneous Lyme disease without neuroborreliosis, for a recommended 21 days with Doxycycline. A fast response to treatment suggests an absence of central neurological involvement. A peripheral neurologic involvement would have been treated with this Doxycycline regimen.^9^

While rare, transient pancytopenia has been described in Lyme disease.^10^ In our patient, the pancytopenia was, indeed, transient. With treatment, all hematologic cell lines returned to normal values.

In *Borrelia burgdorferi* infections, hepatic involvement with mild transaminitis has been reported as common, especially in early disseminated disease with cutaneous damage.^11^ As expected, ALT and AST normalized with antibiotic treatment.

Our patient with an early disseminated Lyme disease presented with multiorgan involvement, making an early diagnosis challenging for hospitalists. The dermatologist’s expertise and knowledge of the different cutaneous presentations of this disease is particularly useful, knowing that Lyme disease cases are now rising in Canada compared to the past decades.^1^ It should also be remembered that an exhaustive history is key to ensuring early and adequate patient care.
